# Draw to Practice: A Qualitative Study Examining Factors Attracting Physicians to Rural Northern Ontario

**DOI:** 10.7759/cureus.55074

**Published:** 2024-02-27

**Authors:** Lily DeMiglio, Jilayne Jolicoeur, Iain R Lamb, Margaret Cousins, Lindsay Nutbrown, Eliseo Orrantia

**Affiliations:** 1 Division of Clinical Sciences, Northern Ontario School of Medicine (NOSM) University, Marathon, CAN; 2 Undergraduate Medical Education (UME), Northern Ontario School of Medicine (NOSM) University, Marathon, CAN; 3 Epidemiology, Marathon Family Health Team, Marathon, CAN; 4 Health Promotion, Marathon Family Health Team, Marathon, CAN

**Keywords:** health human resources, physician recruitment, rural health services, professional practice location, physicians supply & distribution

## Abstract

Introduction

Physician shortages are a persisting issue in rural regions around the world, and rural Northern Ontario, Canada, is no exception. Even with significant government interventions, financial incentives, and rural-specific contracts, physician recruitment to the region remains an ongoing challenge. Refining recruitment strategies based on specific factors that attract physicians to rural practice could help address staffing shortages and, ultimately, enhance healthcare access and outcomes in rural communities. However, the draw to rural practice among physicians is poorly defined. Therefore, this study aims to bridge this knowledge gap and, in doing so, offers insight to better inform recruitment strategies for rural communities.

Methodology

As part of a larger qualitative study on physician retention and recruitment, semi-structured interviews were conducted with 12 physicians who had previously practiced in rural Northern Ontario communities. Interviews captured information about their individual experiences, including perspectives on factors that attracted them to establish a practice in rural Northern Ontario. Transcribed interviews were analyzed to identify recurring themes associated with the factors that affect the decision to practice in rural Northern Ontario.

Results

Participants described the draw to rural practice as being multifactorial and based on overlapping motivations. Key motivations described by participants could be categorized into three broad themes, including rural community connection and exposure, lifestyle and personal preferences, and career considerations. Specifically, participants emphasized the importance of pro-rural mentors and gaining firsthand experience in rural communities as important facilitators that created a connection with these areas. Interest in exploring new parts of the country, alignment with life plans, support of family, and the challenge of rural practice also played pivotal roles in the decision to pursue rural practice. Finally, the opportunity to have a broad scope of practice and serve a need in the healthcare system while receiving fair compensation within the framework of a flexible and supportive contract was also cited as a draw to practice.

Conclusion

The draw to rural practice is multifactorial and based on a wide array of motivations. As a result, recruitment strategies should move beyond single-pronged approaches and recognize the need to design strategies that address the multifaceted motivations and considerations that drive physicians towards rural practice. Designing and implementing recruitment approaches that consider the diverse factors influencing physicians interest in rural career paths is likely to enhance recruitment initiatives and more effectively address shortage of physicians in the region.

## Introduction

Rural Northern Ontario is a historically underserved resource-limited healthcare environment in which health outcomes and life expectancy of residents consistently lag behind provincial and national averages [1,2]. The underlying causes of these disparities can be attributed to a wide range of factors, including, but not limited to, socioeconomic status, income, and education [2]. One significant factor also stems from the limitations of the rural healthcare system, which include a lack of health resources, poor access to healthcare services, and limited specialist care [2]. Among these challenges, insufficient staffing stands out as one of the most significant barriers to accessible, timely, and high-quality care, with shortages being linked to extended wait times, poorer standards of care, and increased strain on the healthcare workforce. Considering the crucial role primary care physicians play in the healthcare system, recruiting more of these providers may be one of the most pragmatic approaches, in the short term, to address the health inequities faced by the more than 250,000 residents in the rural communities across Northern Ontario [3,4]. Yet, even with significant government intervention, including the founding of a medical school with a rural focus (Northern Ontario School of Medicine University, NOSM U), substantive financial incentives, and the introduction of rural-specific contracts designed to attract physicians to rural communities (e.g., the Rural and Northern Physician Group Agreement, RNGPA), the shortage of physicians in this region persists [4-7]. Therefore, improving recruitment approaches may be one of the most easily modifiable strategies to address physician shortages, as most of the modifications to the recruitment process can be implemented at the community level and easily adjusted based on response, compared to systemic-level changes.

Understanding factors that attract physicians to practice is important for improving recruitment efforts as it informs the development of targeted strategies that align with the unique motivations shaping physicians' career choices [8]. To this point, individuals who choose to pursue rural practice have been stereotyped as “missionaries, mercenaries, and/or misfits” [9,10]. This implies physicians are motivated by values similar to those of missionaries, mercenaries, misfits, or a combination of these. In the rural North, there is certainly work among underserved and vulnerable populations, which would appeal to the “missionary” [1,2,9,11]. Similarly, those drawn by financial incentives could be likened to "mercenaries" while the allure of isolated geography, greater autonomy, and less oversight may attract the “misfit” [9,12-14]. However, generalizing the motivations of those seeking rural practice may inadvertently constrain recruitment approaches and, as a result, limit success. While evidence does suggest financial incentives are important, the decision to practice rurally is often influenced by more than just monetary benefits. The opportunity to pursue a broad scope of practice is a notable draw [12,14-16]. Therefore, the motivations to work in rural healthcare may in fact run counter to the adage of “missionaries, mercenaries, and/or misfits.” An overreliance on simplified recruitment strategies informed by this reductionist stereotype may, in part, contribute to the ongoing challenge of rural physician shortages. Therefore, a more detailed and comprehensive exploration of the specific reasons that drive physicians to rural settings is necessary to develop more effective recruitment and retention strategies.

Investing in initiatives to improve recruitment represents a small but significant step toward creating a more sustainable rural healthcare system that can better meet the needs of those living in these communities. Therefore, through qualitative inquiry with physicians who have chosen rural practice in Northern Ontario, this study aimed to gain a deeper understanding of the motivations behind their choice of practice location. Further, considering the shortage of healthcare workers faced by many rural regions worldwide, understanding factors that attract physicians to rural practice holds significant potential. This understanding may provide broadly applicable insights aiding in the development or refinement of more effective recruitment tactics. Such strategies are crucial for enhancing physician recruitment to rural areas, whether in Canada or elsewhere.

## Materials and methods

Ethics approval

Lakehead University (Thunder Bay, Ontario, Canada) Research Ethics Board granted ethics approval for this study (#1467328).

Sampling and recruitment

Eligible participants were primary care physicians who had previously practiced within one of Northern Ontario's RNPGA communities within the past five years. The focus on RNPGA communities was intentional, as these areas exemplify small rural regions within Northern Ontario that face ongoing challenges with physician recruitment. Purposeful sampling was used to identify eligible potential participants, and an invitation to participate in this study was extended to 19 potential participants via phone or email. Those interested were provided with a study introduction letter and a copy of the consent form prior to being interviewed.

Data collection

Between 2019 and 2020, a single researcher (J.J.) conducted 12 one-on-one semi-structured telephone interviews. Interview questions were part of a larger study exploring factors related to the recruitment and retention of physicians in rural Northern Ontario [17]. For this segment of the study, questions were open-ended and designed to elicit participants' perspectives and opinions regarding their motivation to pursue rural practice as well as their experience practicing in rural Northern Ontario. Questions were developed by the research team and primarily informed through a scan of academic literature, available on PubMed, that focused on factors proven to impact physician recruitment and retention [4,7,11,14,18].

Data analysis and analytical framework

This study employed a hermeneutic phenomenological approach to gain deeper insights into the factors that attract physicians to practice medicine in rural Northern Ontario. The objective was to capture participants' lived experiences, providing a descriptive account of their perspectives and realities to identify the commonalities that underly their motivations to practice in the rural North.

Interviews were audio recorded using TapeACall (Teltech Systems, Inc., New York, NY). All 12 interviews were transcribed verbatim by a member of the research team (L.N.). The accuracy of the transcription was verified against the original recording by another member of the research team (J.J.). Transcripts were then uploaded to Dedoose (v9.0, SocioCultural Research Consultants LLC, Los Angeles, CA), a program for managing, analyzing, and presenting qualitative and mixed-method research data. Interview transcripts were reviewed by four research members (E.O., L.D., J.J., and L.N.). Following transcription, the researchers conducted thematic analysis as guided by Burnard [19] and Braun and Clarke [20]. In brief, the research team familiarized themselves with each transcript and created preliminary codes to capture phrases or sentences conveying distinct ideas expressed by participants. Through regular meetings, these codes were discussed, compared, and organized into common themes, which were eventually integrated into broader, overarching themes. Thematic saturation was considered once the team determined that no new themes were emerging from the data, indicating a comprehensive capture of the thematic elements present in the interviews.

## Results

Participant demographic and professional characteristics

Out of the physicians who were contacted, 63% (12/19) agreed to participate in the interviews. Participant demographics and characteristics are detailed in Table 1.

**Table 1:** Participant demographic and professional characteristics

Participant demographic and professional characteristics		Response
Gender of participants	Male	75% (9/12)
	Female	25% (3/12)
Practice location		11 of the 38 RNGPA within Northern Ontario
Upbringing in Northern Ontario		8% (1/12)
Years in rural community		1 to 27 years
Registration with provincial college	Registered	92% (11/12)
	Resigned registration	8% (1/12)
Medical school education	Ontario	50% (6/12)
	Elsewhere in Canada	42% (5/12)
	Outside of the Canada	8% (1/12)
Time since completion of residency	Within the last 5 years	8% (1/12)
	Within the last 10-15 years	50% (6/12)
	Within the last 20-25 years	17% (2/12)
	Within the last 30-35 years	8% (1/12)
	Within the 35-40 years	8% (1/12)

Draw to rural practice

The analysis uncovered common elements that influenced physicians' attraction to rural practice in Northern Ontario. These elements could be categorized into three overarching themes: rural community connection and exposure; lifestyle and personal preferences; and career considerations.

Rural community connection and exposure 

The draw towards pursue rural practice was shaped, in part, by experiences with and ties to rural communities. This came in diverse forms, such as having personal and/or professional connections working in rural areas, previous training experiences in rural settings (e.g., during medical school or residency), prior locum practice within rural communities, and visits to the area. While the type of exposure to rural environments was varied, these touchpoints were all recognized as providing valuable insight into the unique aspects of rural medicine, thus playing an important role in the decision to pursue rural practice. Interview excerpts that best illustrate the elements are presented.

Personal and Professional Connections

Physicians were often drawn to rural practice if they had connections such as a friend, colleague, or preceptor already established in that community. For example, one of the RNPGA communities had success recruiting new physicians from a particular university because of the influence of a preceptor who taught at the university but had also worked in a rural community:

“He would tell us all about his experiences in the North and what it was like. So that got a lot of people interested so a lot of my colleagues or fellow residents went up there with him on electives.”

- Participant 5

Rural Training

Family physicians discussed that having previous rural experience during medical school or their residency made the prospect of practicing rurally more appealing. Participants confirmed they often had educational experiences in rural locations prior to beginning their practice:

“An experience as a medical resident. Well actually both as a medical student and a medical resident. I just loved the practice in rural Northern Ontario. I really like [emergency medicine], loved the community. It was really just having really good experiences as a medical learner.”

- Participant 3

Locum Experience

The Rural Family Medicine Locum Program (RFMLP) provides physicians with the opportunity to work in rural communities in Ontario where coverage is needed. Participation in the RFMLP is common amongst physicians, as the program provides ample financial incentive for those coming out of residency and allows physicians to become familiar with a community both personally and professionally before committing to the RNPGA contract. It was common for participants to have completed a locum period in an RNPGA community prior to beginning their practice there:

“I first did some locums there and they developed a need. And so we agreed to sign a contract.”

- Participant 12

Community Visit

Community visits were also considered an important component in choosing a location to begin a career in rural family medicine. A participant highlighted their positive experience as follows:

“My husband and I went up to those two places and toured around and I had such an amazing experience with the [community] hospital and with the physicians there, they were very welcoming. The physician and her husband were there and her husband made an extra double effort to make my husband feel special and at home. And then when I met the doctor I would be working with at the clinic, which was a different doctor, we had a really nice rapport and so I pretty much made my decision right then that that’s where I wanted to go and they were so happy to have me.”

- Participant 5

Lifestyle and personal considerations

Decisions to practice in rural communities were also shaped by a combination of personal and lifestyle considerations, highlighting the importance of these factors in influencing the appeal of rural practice, as noted elsewhere [14,21,22,23]. Both the prospect of exploring new parts of the country and the potential for personal and professional growth through the challenges of working in rural regions were cited as appealing. Moreover, respondents found appeal in the fact that the professional demands of rural practice aligned with their present life stage or future plans. Family support and their fulfillment in the community were also highlighted as important factors.

Northern Interest

Northern Ontario's vast geography and proximity to nature were the key attractions. One participant shared their motivation for relocating to Northern Ontario as follows:

“Having that opportunity to experience absolute wilderness and beauty and that’s what brought us here in the first place.”

- Participant 7

Character Traits

Participants expressed interest in rural practice because it would be an interesting or challenging experience as a new graduate. In particular, a participant discussed that an appeal was the degree of uncertainty they may face as a rural physician:

“I wanted to push my boundaries like delivering babies and intubating and the scary things that we didn’t get to do in residency very often. So I spent the next 6 months before I graduated filling all the gaps. So I did extra emerge and I did extra OB and I really pushed my comfort zone. And I let all my preceptors know in advance that that was my goal to be independent and I would be alone in very few months doing it by myself so my last bit of residency was probably more intense than the average just because they wanted to have me ready.”

- Participant 5

Life Plan

Some participants indicated that relocating to rural Ontario to start their practice was part of their intended life plan. The factors that attracted physicians to rural practice included the financial feasibility of repaying student loans, the desire to return to a small town, and the aspiration to raise a family in a rural environment.

“I have three children and it was really so much easier to raise them in a small community than it was to raise them in a big city. So that was actually one of the biggest draws. The ease of childcare, when you are working 24 hours a day or are on call 24 hours. It was so much easier than you know being in the city and trying to get them in daycare. There were so many more opportunities, so much better school access, it was overall just a much better experience for young children to have.”

- Participant 3

Family Support

Having the support of a spouse and family played a crucial role in the decision to begin rural practice. Failure to 'recruit' the spouse or partner of the physician often led to difficulties in recruiting and retaining the physician [18]. A participant highlighted the importance of spousal support and advised those looking to practice rurally to consider their partner or spouse’s employment opportunities and personal satisfaction when selecting a community:

“Ensure that your partner has an equally fulfilling role in the community. Whether it is work or something else, that is the ultimate retention device. . .if you don’t retain the partner, and the family of the physician, you are going to lose the physician.”

- Participant 2

Career considerations

The diverse elements of a career in rural practice were recognized as key factors in attracting physicians to Northern Ontario. These elements included the breadth of scope of practice, an attractive remuneration package, favorable contractual agreements, and a sense of fulfillment in meeting the healthcare needs of underserved communities. Consequently, rural practice emerged as a compelling and rewarding pursuit for many physicians.

Rural Generalist Practice

The “Rural Generalist” scope is typically wide-ranging, covering areas such as primary care, acute care, and hospital inpatient care work. Physicians expressed a preference for engaging in a comprehensive scope of practice that is not as readily available in urban settings.

“The thing that really drew me up there was the broader scope and being able to do and have your hand in emergent family clinic-based medicine. long-term care, inpatient care, palliative care, homecare, really being able to do [it] all.”

- Participant 1

The RNPGA Contract

The RNPGA is a unique contract that allows physicians more freedom regarding their practice management. The contract typically offers competitive compensation, flexibility, and locum coverage, giving the physician significant time away from practice for continuing medical education or vacation. One participant described this as a more “holistic” practice.

“I think financially it wasn’t so much the amount of money that you could make so much as that’s the way you were paid that allowed you to practice. So it was more kind of holistic, you could do a full spectrum practice and still make a decent salary. I don’t know I was looking to make more, but it let you do more things.”

- Participant 11

Fulfilling a Health System Need

Persistent workforce challenges in Northern Ontario's rural healthcare system frequently result in unfilled positions, leaving communities unable to recruit and retain a sufficient number of physicians needed to support their population. As a result, physicians wanted to fulfill a need in the healthcare system.

“Incredible opportunity to really revitalize healthcare from a leadership position so … you have these abilities to be leaders and chief-of-staff- and really just change the entire healthcare delivery systems and structures of healthcare delivery in small communities. And you can change things for the better, improving physician accountability, improving patient outcomes, you can advocate for changes on a political level, a bigger level, a structural level....”

- Participant 3

Financial Incentives

Practicing in rural Northern Ontario often comes with highly competitive remuneration packages and financial incentives. This was a drawing factor consistently mentioned by participants when discussing the appealing features of rural practice. One participant explained the appeal of financial incentives to offset student debt:

“One was the potential income... In areas of need the financial reimbursement is always higher and I wasn’t too far out of finishing residency so student loans, line of credit, I still had all of those to deal with so it was a good opportunity to pay all that off.”

- Participant 6

## Discussion

While the "mercenary, missionary, misfit" trope may capture some aspects of the motivations for rural practice, our findings suggest it oversimplifies the diverse and multifaceted reasons to pursue rural practice. We did find those attracted to practice in rural Northern Ontario exhibited a missionary-like desire to meet the needs of the underserved. Further, the prospect of higher remuneration does serve as a draw to practice mirroring the “mercenary" motivation. However, there was no evidence of attraction for the "misfit" seeking to evade confirmation of the expectations and standards of care imposed by colleagues or regulating bodies. Rather, the draw to practice is multifaceted, based on overlapping experiences and motivations that include exposure to rural environments, the compatibility of rural lifestyles with personal values and life stages, and considerations related to career development.

The significance of rural community connection and exposure

Rural exposure in both educational and professional contexts was a foundational experience that played a significant role in influencing participants' decisions to pursue rural practice. This further emphasizes the importance of early and ongoing exposure to rural environments within medical education and professional training programmes [24]. This stands to enhance rural exposure for medical learners, offering them the opportunity to experience both the challenges and rewards of rural practice to help foster a deeper understanding of rural healthcare needs, particularly among those who may not have sought these opportunities otherwise [24]. Facilitating this increased awareness among physicians may lead them to consider a career path that involves rural practice. Further, establishing programmes that offer well-compensated opportunities for current medical professionals to engage in locum placements within rural communities, similar to Ontario's RFMLP, may serve as an effective strategy for both new and established physicians to gain experience and exposure to rural practice. These opportunities may increase interest in addressing healthcare disparities in these communities and help foster a commitment to advancing care in underserved areas.

Exposure to rural environments played a foundational role for those who chose to pursue rural practice. We found this extends beyond practical experience to the substantial impact mentors and preceptors with rural clinical experience can have on shaping the decision to pursue experiences in rural healthcare. Given the significance of these "pro-rural" mentors, medical education programs should provide medical learners with opportunities for mentorship from rural practitioners. In Ontario, the impact of rural influences is acknowledged through mentorship programs facilitated by the Society of Rural Physicians of Canada (SRPC), with similar rural-specific mentorship initiatives being implemented in other regions as well [25,26].

Data indicate medical learners are more likely to establish their practice in the regions where they were raised [27]. This has led some medical schools to implement preferential admission criteria for rural candidates with the aim of graduating physicians who have an "innate" draw to rural practice [7]. Interestingly, the majority of our respondents were not originally from rural Northern Ontario; however, each had prior educational or professional experiences in rural communities that they cited as playing an important role in influencing their decision to pursue rural practice. This suggests that having a rural upbringing may only be one factor influencing the draw to practice and underscores the role that experiences in rural communities play in the professional development and career choices of physicians. This, in turn, further highlights the importance of providing opportunities for individuals to gain exposure to rural environments, whether through direct experience or mentorship, particularly within professional and educational contexts.

Lifestyle and professional considerations as factors to practicing in rural Northern Ontario

While some may consider rural living a disadvantage, others gravitate towards it for its lifestyle. Our findings show those drawn to rural practice embraced the sense of adventure it offers, while others saw the move to a rural area as an alignment with their life plan, whether it be the desire to return to a small town or the aspiration to raise a family in a rural setting. Given that potential recruits may be deterred by the lack of amenities, communities actively recruiting physicians should emphasize the distinct lifestyle advantages that rural living can offer [28]. This may include shorter commutes, low crime rates, access to natural environments, and a sense of tranquility. This allure can be particularly appealing to those valuing a more serene way of living or wanting a change from the fast-paced and often stress-inducing urban environment.

While it may seem somewhat obvious, respondents highlighted the significance of their spouse/partner and/or family member’s fulfillment when contemplating rural practice. This aligns with findings that have shown physicians are more likely to leave rural practice when their partners express dissatisfaction with their own careers [17]. As a result, communities should recognize the significance of recruitment visits and invest time and effort to ensure that potential recruits' partners and/or families have positive experiences that showcase the unique advantages of rural life from their perspective. Furthermore, expressing openness to accommodate or consider the partner's career can be pivotal in demonstrating a commitment to supporting the well-being and contentment of the entire family unit.

Respondents also cited that an appealing aspect of rural practice was the opportunity for personal/professional growth and development that arises from the challenges of delivering care in the resource-limited environment of rural Northern Ontario. Considering this, highlighting the rewards of overcoming these challenges may be beneficial in attracting individuals who possess a strong desire to confront, learn from, and develop through such experiences to become a more resilient practitioner.

Importance of career considerations in the pursuit of rural practice

The lack of health human resources in rural Northern Ontario, especially the limited access to specialist care, has led to the emergence of the "rural generalist." This describes physicians in rural areas who provide comprehensive medical care, covering a broad spectrum of medical disciplines to meet community needs [29]. Our study shows that the opportunity to practice and be exposed to a wider range of medical disciplines was an appealing aspect of rural practice. Therefore, highlighting the diverse practice opportunities available in rural healthcare settings and how such environments can enrich the professional lives of practitioners by immersion in varied clinical landscape may serve as a key strategy in the recruitment of healthcare professionals.

In our study, individuals with a strong sense of social justice were dedicated to serving residents of Northern Ontario, a population that faces longstanding and historical health inequities when compared to the province’s urban populations. While idealism and a desire to fulfill a need in the healthcare system may draw new recruits to the rural North, it is important to provide ongoing support to retain them over the long term. Regardless of altruistic motivations, the aspect of financial compensation remains a key factor. Participants were enticed by the rural-specific contract that exists in Northern Ontario (RNPGA), which offers competitive compensation alongside a flexible schedule and locum support for breaks in practice. This is in sharp contrast to the fee-for-service practice environment, which presents more barriers and challenges for physicians seeking a more favorable work-life balance [30]. Having a contract such as the RNPGA allows for certainty around compensation and vacation days, which is attractive for many. Therefore, the development of specific contracts that explicitly outline occupational expectations and compensation could serve as an effective approach for recruitment.

Translatability of findings

In many ways, rural Northern Ontario exemplifies the archetypal rural health setting. These communities are often geographically isolated from large metropolitan centres, have limited access to healthcare providers and specialists, and have a shortage of resources [1,2,31]. Moreover, these communities are affected by socioeconomic challenges and various other factors that impact the social determinants of health, leading to a population that faces poorer health outcomes compared to their urban counterparts [1,2,31]. Recognizing the similarity rural Northern Ontario shares with other rural regions, the motivation to practice in such environments may also share similarities. However, the extent of these similarities and their implication for physician recruitment and retention in rural settings remain widely unknown and merits future investigation.

Limitations

It is important to recognize that draws to practice may differ depending on location. Our findings only capture the opinions of primary care physicians representing 11 of the 38 RNGA communities providing primary care services in rural Northern Ontario [32]. Therefore, the applicability of these findings to the broader rural North as well as other rural health regions in Canada and abroad may be limited. Furthermore, with only 11 communities represented, distinct draws to practice unique to certain communities within Northern Ontario may not have been captured. Another limitation of this study is that we interviewed only one provider in 10 out of the 11 communities; therefore, our findings may not cover all the specific reasons for practicing in rural areas, including those unique to the communities represented in this study. As our study exclusively interviewed primary care physicians, it may not reflect the factors influencing the decisions of other healthcare professionals to work in rural settings. Therefore, future studies aiming to better understand the factors that attract healthcare professionals to rural practice in Northern Ontario may benefit from including a more diverse array of rural Northern communities and involving a broader range of healthcare providers. Another challenge was comprehending the relative importance each factor had on the provider's decision to pursue rural practice. This makes it challenging to prioritize recommendations, which may be important for rural communities with limited capacity to implement recruitment strategies. Further, considering the retrospective nature of this study, another limitation is the reliance on the participants' recollections about what attracted them to rural practice, which introduces the possibility of recall bias. Despite these limitations, our study contributes insights into some of the factors influencing physicians to practice in rural Northern Ontario, serving as a foundation for further inquiry.

## Conclusions

The stereotype that physicians drawn to rural practice fall into the archetype of “missionary,” "mercenary,” and/or “misfit” oversimplifies and overlooks the intricate and diverse motivations that underlie the complex nature of pursuing rural practice. Instead, our study finds the draw to rural practice is multifaceted and is shaped by factors such as exposure to rural environments, the alignment of rural practice with lifestyle and life plans, and considerations/aspirations related to one's career. As a result, recruitment strategies should move beyond single-pronged approaches and instead recognize the need to design strategies that incorporate the multifaceted motivations and considerations that drive physicians towards rural practice. This approach will likely translate to a targeted result in recruitment strategies that better align with the wide range of factors influencing physicians' choices to work in rural locations, which, in turn, may assist in mitigating the physician shortage in these areas.
